# Salivary Interferon Gamma and Interleukin-4 Levels in Patients Suffering from Oral Lichen Planus

**DOI:** 10.22074/cellj.2015.16

**Published:** 2015-10-07

**Authors:** Hossein Malekzadeh, Maryam Robati, Hojatollah Yousefimanesh, Mehri Ghafourian Boroujerdnia, Reza Nadripour

**Affiliations:** 1 Department of Oral Medicine, Faculty of Dentistry, Ahvaz Jundishapur University of Medical Sciences, Ahvaz, Iran; 2 Department of Periodontics, Faculty of Dentistry, Ahvaz Jundishapur University of Medical Sciences, Ahvaz, Iran; 3 Department of Immunology, Faculty of Medicine, Ahvaz Jundishapur University of Medical Sciences, Ahvaz, Iran

**Keywords:** Lichen Planus, Interleukin-4, Saliva, Interferon Gamma

## Abstract

**Objective:**

Oral lichen planus (OLP) is a chronic inflammatory disease. Immunological factor may act as etiological factor. The cellular immune cells such as T cells are impor- tant in pathogenesis. Interferon gamma (IFN-γ) and interleukin 4 (IL-4) are secreted by T-helper 1 (Th1) and Th2, respectively. The aim of this study was to investigate the cor- relation between salivary levels of IFN-γ and IL-4 with OLP.

**Materials and Methods:**

This case control study included sixty three Iranian OLP patients who were selected from the Department of Oral Medicine of Ahvaz Jundishapur University of Medical Sciences from January to July 2013. An equal number of healthy volunteers were also selected as a control group. The OLP patients were then divided into two follow- ing sub-groups: reticular (n=30) and erythematous/ulcerative (n=33). All patients had no systemic disease and received no medication. IFN-γ and IL-4 levels in whole unstimulated saliva (WUS) were measured using the enzyme-linked immunosorbent assay (ELISA) test. Data analysis was done using t test, ANOVA, least significant difference (LSD) test, and the Kruskal-Wallis test.

**Results:**

Reticular OLP patients showed higher salivary IFN-γ (7.74 ± 0.09 pg/ml ) and IL-4 (3.876 ± 0.05 pg/ml) levels compared with the control group, indicating that difference was significant. Salivary IFN-γ/IL-4 ratio significantly increased compared with control group (P=0.042). Salivary IFN-γ and IL-4 levels between sub-groups (re- ticular and erythematous/ulcerative) were not significantly different (2.6 ± 0.06 and 2.3 ± 0.05, respectively, P<0.05).

**Conclusion:**

Salivary IFN-γ and IL-4 levels were increased in OLP patients. An increase of salivary IFN-γ/IL-4 ratio in OLP patients showed that Th1 might have a dominant role in the OLP pathogenesis.

## Introduction

Oral lichen planus (OLP) is a chronic inflammatory disease that affects 1-2% of the adult population (1). Clinical features of OLP lesions are white plaque, erythema, ulcer, and blister (2). This disease is more likely to occur in female than male (3). The buccal mucosa, tongue and gingiva are involved intraoral site in OLP, whereas intraoral site is involved occasionally (4). The local reaction of OLP may be due to cytokine production by lymphocyte (5). Although the etiology of OLP is unknown, it seems that this disease is T cells-mediated autoimmune disease in which CD8^+^ T cells trigger apoptosis of oral epithelial cells (6). 

The T-helper (Th) cells based on cytokine production are classified into Th1 cell and Th2 cell subtypes. The Th1 cells secrete interferon gamma (IFN-γ), interleukin 2 (IL-2) and tumor necrosis factor alpha (TNF-α), while Th2 cells secrete IL-4, IL-5, IL-10 and IL-13 (7). Therefore, IFN-γ and IL-4 are representatives of cytokines which are produced by Th1 and Th2 cells, respectively (8). 

IFN-γ is a soluble dimer cytokine that is also named macrophage activating factor (9). This cytokine has an important role in adaptive and innate immunity, especially against viral infection, intracellular bacteria and tumor controlling (10). In terms of biology, IFN-γ enhances the activity of natural killer (NK) cells, lysosomal function in macrophage, adhesion of leukocyte cells during intracellular migration and intracellular defence factors (11). 

IL-4 induces differentiation of T cell into Th_2_ and it seems that basophil cells play a role in this process (12). Furthermore IL-4 plays a role in B cells and T cell activation, adaptive humoral immune response and pathological inflammation reduction (13). It has been demonstrated that the Th1/Th2 cell imbalance is involved in pathogenesis and development of many kinds of autoimmune disorders such as Behcet’s disease (14). Since OLP has an immunological base, it seems that cytokine profiles change frequently during this disorder (15). 

In a study by Khan et al. (16), after evaluating cytokine presentation in OLP, they did not find IL-4 secretion in OLP lesion and concluded that T cells were not able to produce cytokines. In another study by Yamamoto and Osaki (17) and Yamamoto et al. (18), they demonstrated that IL-4 production significantly increased in tissue and serum of OLP patients. However, there is a discrepancy about IFN-γ level between mentioned-studies. One study demonstrated a significant increase of IFN-γ level between OLP patients and control group (19). Another controversial study showed that salivary IFN-γ level significantly decreased as compared with control group (20). A number of studies suggested that salivary sample compared to serum sample might be a more sensitive method to reflect the cytokine production (21). 

OLP has a high prevalence in Iran and there was no agreement about its pathogenesis; therefore, this study was designed to investigate the expression levels of salivary IFN-γ and IL-4 produced by Th1 and Th2 cells, respectively. 

## Materials and Methods

This case control study included sixty three Iranian patients with clinical and histopathological diagnosis of OLP. The case group (n=63) was then divided into two following sub-groups: erythematous/ulcerative (n=33) and reticular (n=30). Sixty three ageand sex-matched healthy volunteers were selected as a control group. The oral biopsy confirmed the clinical diagnosis of OLP in both sub-groups. 

All case and control groups had no history of smoking, systemic disorders (such as diabetes mellitus, chronic hepatitis, etc.), and treatments for OLP during the past three months. The pregnant and lactating women were excluded form study. The Ethics Committee of the Ahvaz Jundishapur University of Medical Sciences approved this study according the 59^th^World Medical Association (WMA) General Assembly, Seoul, Korea, October 2008. All participants signed an informed consent form before taking a part in this study. 

### Sample collection

All patients were selected from the Department of Oral Medicine of Ahvaz Jundishapur University of Medical Sciences from January to July 2013. The purpose of the study was explained to all patients before sampling. Whole unstimulated saliva (WUS) samples were collected using standard technique described by Navazesh (22). In brief, the patients were asked to refrain from eating and drinking one hour before sampling. All patients were requested to swallow and then spit into a sterile tube. After gathering about 5 ml of saliva, it was transferred to the immunology laboratory and centrifuged (Hettich, Germany) at 3500 g for 20 minutes. The supernatants were separated and frozen at -20˚C until sampling was completed. All saliva sampling was done between 8:00 a.m. to 10:00 a.m. to avoid circadian variations. 

### Cytokine assay

After completing saliva sampling, the salivary IFN-γ and IL-4 concentrations were determined using the commercial enzyme-linked immunosorbent assay (ELISA) kit (eBioscience, San Diego, USA). 

### Statistical analysis

Data analysis was performed using SPSS (SPSS Inc., Chicago, IL, USA) version 19 software. The Kolmogorov–Smirnov test was used to determine the normality of distribution. For comparison of IFN-γ level between all cases and between supgroups with control group, t test and Kruskal – Wallis test were used, respectively. For comparison of IL-4 level between all cases and between sup-groups with the control group, ANOVA and least significant difference (LSD) test were used, respectively. The P<0.05 was significant.

## Results

This study contained 126 patients that were divided into two sub-groups (case) and a control group. The control group included 47.6 and 52.4% male and female, respectively. The mean age values of case and control group were 41.5 ± 0.4 and 37 ± 0.6, respectively, indicating that there was no significant difference (P=0.87). Table 1 shows demographic data for case and control groups separately.

**Table 1:** Demographic data of case and control groups


Groups	Age (Y)	Gender (n) %		Total
		Male	Female	

Erythematous/ulcerative	41 ± 0.1	(13) 38%	(20) 62%	33
Reticular	41 ± 0.8	(12) 40%	(18) 60%	30
Control	37 ± 0.6	(30) 47.6%	(33) 52.4%	63


The salivary IFN-γ level of case group significantly increased compared with control group (P=0.041). The cytokine levels in erythematous/ ulcerative and reticular sub-groups were 8.04 ± 0.08 pg/ml and 7.74 ± 0.09 pg/ml, respectively, suggesting that there was a significant difference as compared to the control group (1.69 ± 0.01 pg/ ml, P<0.5). In addition, there was no significant difference of the salivary IFN-γ level between two subgroups (P=0.124). The salivary IL4 level in case group was significantly increased compared with control group (P=0.043). The salivary IL-4 levels in reticular and erythematous/ulcerative sub-groups (4.582 ± 0.03 pg/ml and 3.876 ± 0.05 pg/ml, respectively) were significantly higher than those in the control group (1.58 ± 0.02, P<0.05). There was no significant difference regarding the IL-4 levels between two sub-group (P<0.05) (Table 1).

**Fig.1:**
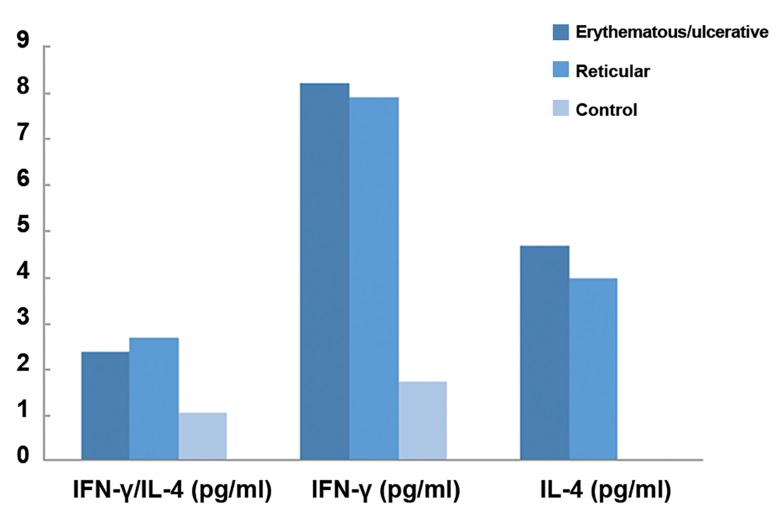
The expression levels of IFN-γ and IL-4 in case and control groups. IFN; Interferon and IL; Interleukin.

The salivary IFN-γ/IL-4 ratio of case group significantly increased compared with control group, indicating that there was no significant difference regarding IFN-γ/IL-4 ratio levels between sub-group (P>0.05). However, there was a significant difference regarding IFN-γ/IL-4 ratio levels between erythematous/ulcerative (2.3 ± 0.05 pg/ml) and reticular sub-groups (2.6 ± 0.06 pg/ml) with control group (1.01 ± 0.03 pg/ ml, P<0.05) (Fig .1).

## Discussion

Better understanding of autoimmune features of OLP leads to develop an effective method to control this disease, so this study designed to investigate the correlation between salivary levels of IFN-γ and IL-4 with OLP. IFN-γ and IL-4 levels are considered to be the important cytokines being produced by Th1 cell and Th2 cells, respectively (23). Khan et al. (16) demonstrated unstimulated T cells in OLP lesions; however, they failed to find secretion of IL-4, IL-10 and transforming growth factor beta (TGF-β). We found IL-4 expression in our study which is likely due to different method used because their study was *in vitro*, but our study was *in vivo*. Furthermore our study demonstrated that IFN-γ and IL-4 levels increased compared to control group.

On the contrary, Tao et al. (19) found no significant difference between IFN-γ and IL-4 levels in whole OLP with control groups. We included a large population in this study and used a high sensitivity ELISA kit that explains such conflicting findings with Tao et al.’s study. Nevertheless, both mentionedstudies detected IFN-γ and IL-4 levels in saliva and confirmed the immunologic base of OLP.

In a study by Liu et al. (20, 24), they demonstrated that salivary IFN-γ level significantly decreased compared with control group, but salivary IL-4 level in OLP group increased compared with control group. We found that these two biomarkers increased significantly in OLP patients compared with the control group. It is noteworthy to mention that some studies concluded that IFN-γ level and OLP development were both influenced by genetic polymorphism (25, 26).

Yamamoto and Osaki (17) and Yamamoto et al. (18) demonstrated a significant increase in the number of IL-4 cells in the OLP tissue specimen and a slight increase of serum IL-4 in OLP patients. Their findings were consistent with our results, suggesting that OLP affects local site and influences cytokine secretion. Zhang et al. (21) upheld this claim and suggested that disease-related cytokine production might be reflected to be more sensitive in saliva compared to serum. Local cytokine production by inflammatory cells and/or by epithelial cells increased salivary cytokine (27).

Our findings also showed that the salivary IFN-γ/ IL-4 ratio significantly increased compared to control group. The IFN-γ concentration in erythematous/ ulcerative and reticular sub-groups increased nine and twelve times, respectively, more than control group. This result may suggest that Th1 cell is more dominant than Th2 cell. Many studies revealed the same result (28, 29), although this conclusion did not supported by Rhodus et al. (30). They collected from lesion tissue transudates and detected cytokines, but we detected and evaluated them in saliva. The different race and type (saliva and tissue) of sampling may explain these conflicting findings.

This study suggested that Th1 cell was dominant in cytokine secretion, but does not mean that Th1 cell is causative factor and responsible for Th1/ Th2 cell imbalance. Since there is a correlation between the polymorphism and cytokine secretion (31), such findings could be seen. Therefore, it is required to compare the level of these factors in both saliva and serum and to consider polymorphism as one of key indicators.

## Conclusion

The saliva is safe and non invasive sample for evaluation of OLP. Imbalance between Th1/Th2 cells may influence OLP pathogenesis and cause an increase in concentrations of IFN-γ and IL-4 in OLP patients.
